# Myocardial lipid remodeling in patients with myocardial infarction undergoing cardiac surgery

**DOI:** 10.1016/j.jmccpl.2026.100875

**Published:** 2026-10-07

**Authors:** Tomoyuki Shiozawa, Tomoyasu Kadoguchi, Hyeon-Cheol Lee-Okada, Kazunori Shimada, Satoshi Matsushita, Takehiko Yokomizo, Tetsuro Miyazaki, Tatsuro Aikawa, Hidemori Hayashi, Satoru Suwa, Atsushi Amano, Yuji Nakazato, Hiroyuki Daida, Minoru Tabata, Naoshi Fukui, Nobuho Tanaka, Hirotaka Tsuno, Tohru Minamino

**Affiliations:** a Department of Cardiovascular Medicine, Juntendo University Shizuoka Hospital, Shizuoka, Japan; b Department of Life Sciences, Graduate School of Arts and Sciences, The University of Tokyo, Tokyo, Japan; c Sportology Center, Juntendo University Graduate School of Medicine, Tokyo, Japan; d Department of Biochemistry, Juntendo University Graduate School of Medicine, Tokyo, Japan; e Department of Cardiovascular Biology and Medicine, Juntendo University Graduate School of Medicine, Tokyo, Japan; f Department of Cardiovascular Surgery, Juntendo University Graduate School of Medicine, Tokyo, Japan; g Laboratory of Bioregulatory Clinical Pharmacology, Faculty of Pharmacy, Juntendo University, Chiba, Japan; h Clinical Research Center, National Hospital Organization Sagamihara National Hospital, Sagamihara, Japan; i Faculty of Health Science, Juntendo University, Tokyo, Japan

**Keywords:** Phospholipid, Fatty acid, Myocardial tissue, Lipidomics, Myocardial infarction

## Abstract

Myocardial lipid remodeling in myocardial infarction (MI) remains insufficiently characterized in human myocardium. Using liquid chromatography–mass spectrometry, we analyzed left atrial appendage tissue from patients with and without MI undergoing cardiac surgery. MI was characterized by reduced phospholipid species containing omega-3 polyunsaturated fatty acids (PUFAs) and myristic acid, together with reduced diacylglycerol species containing omega-3 PUFAs. Reduced phosphatidylcholine, phosphatidylethanolamine, phosphatidylinositol, and plasmalogens were observed, whereas triacylglycerols were enriched. Loss of polyunsaturated phospholipids and accumulation of relatively saturated triacylglycerols indicate a shift toward neutral lipid storage in human MI myocardium.

## Highlights

• LC–MS lipidomics of human LAA tissue was performed in myocardial infarction (MI) vs. non-MI.
• MI tissue showed triacylglycerol accumulation and phospholipid depletion.
• Phospholipid species containing omega-3 PUFAs were significantly reduced in MI.
• Lipid chain patterns suggested a shift toward neutral lipid storage in MI.

## Graphical abstract

**Figure:**
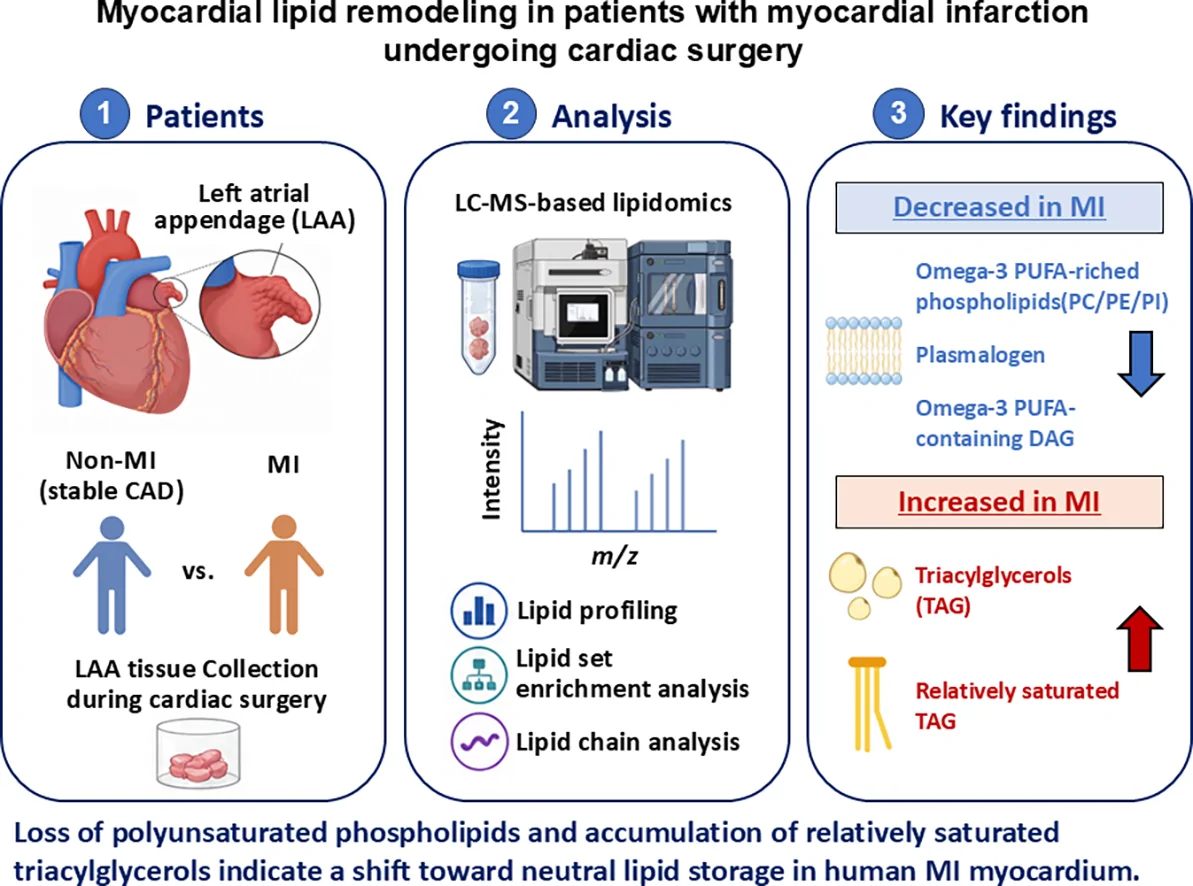

## 1 Introduction

Myocardial infarction (MI) is the most acute manifestation of coronary artery disease and a leading cause of morbidity and mortality worldwide. Although circulating lipid profiles, cardiac biomarkers, and imaging modalities are widely used for diagnosis and risk stratification, they do not fully reflect metabolic alterations occurring within the myocardium during disease progression.

Mass spectrometry (MS) –based lipidomics enables comprehensive characterization of lipid species and has been widely applied to plasma or serum samples for cardiovascular risk prediction [1], [2]. However, lipid remodeling within myocardial tissue itself remains poorly defined. Because myocardial lipid metabolism plays a central role in cardiac energy homeostasis and dysfunction, direct tissue-level lipid profiling may provide insights beyond circulating biomarkers.

Here, we performed liquid chromatography–MS (LC–MS)–based lipidomic profiling of left atrial appendage (LAA) tissue obtained from patients with and without MI undergoing cardiac surgery to characterize MI-associated myocardial lipid alterations.

## 2 Materials and methods

We retrospectively analyzed 28 consecutive patients who underwent off-pump coronary artery bypass grafting (CABG) with routine LAA dissection at Juntendo University Hospital between September 2016 and March 2017. Individuals with prior open-heart surgery or a history of chest irradiation were excluded. Patients were clinically classified according to the presence or absence of a prior diagnosis of myocardial infarction; the non-MI group consisted of patients with stable coronary artery disease without a prior diagnosis of myocardial infarction. The clinical characteristics and laboratory data presented in Supplemental Table 1 were obtained at the time of preoperative hospitalization, before surgery. Therefore, these data represent the patients' baseline clinical status prior to surgery. LAA tissue was collected intraoperatively during cardiac surgery. Propensity score matching based on age and sex using EZR software (Easy R version 1.27) yielded 14 patients (7 per group) for analysis. The Ethics Committee of Juntendo University approved this study. This study was managed according to the principles of the Declaration of Helsinki (approval number: H15-147). Written informed consent was obtained from all participants before enrollment in the study.

LAA tissue samples were immediately preserved in cold cardioplegic solution and stored at −80 °C until analysis. Semi-quantitative Lipidomic profiling was performed using LC–MS as previously described [3], and the data were normalized using internal standards and wet weight of the tissue and expressed as relative quantity. Briefly, lipids were extracted using the Bligh and Dyer method and analyzed by ultra-high-performance LC–MS, and lipid species were quantified using multiple reaction monitoring.

Baseline comparisons between the MI and non-MI groups were performed using the Shapiro–Wilk test to assess normality, followed by unpaired *t*-tests for normally distributed variables and Mann–Whitney *U* tests for non-normally distributed variables. Categorical variables were compared using Pearson's chi-square test. These analyses were performed using JMP software (SAS Institute, Cary, NC, USA), with a *p*-value <0.05 considered statistically significant. Lipidomic analyses, including orthogonal partial least squares–discriminant analysis (OPLS-DA), lipid set enrichment analysis (LSEA), and lipid chain analysis, were conducted using R (version 4.4.2) and the lipidr package (version 2.20.0) [4]. LSEA was performed using fgsea with individual lipid species ranked by log2 fold-change (logFC), and class-level significance was defined as a Benjamini–Hochberg false discovery rate (FDR) < 0.05.

## 3 Results and discussion

Baseline characteristics after propensity score matching are summarized in Supplemental Table 1. Statin use was significantly lower and plasma triglyceride (TG) levels were higher in the MI group (both *p* < 0.05).

Wide-target lipidomics analysis identified 18 lipid classes in LAA tissue (Supplementary dataset). OPLS-DA demonstrated clear separation between MI and non-MI groups, indicating distinct myocardial lipid profiles (Fig. 1A). Loading plots revealed clustering of triacylglycerol (TAG) species toward the MI group. Among the top 20 differentially abundant lipids, phospholipid species containing omega-3 polyunsaturated fatty acids (PUFAs), including eicosapentaenoic acid (EPA; C20:5) and docosahexaenoic acid (DHA; C22:6), as well as phospholipids species containing myristic-acid (C14:0), and diacylglycerols species containing omega-3 PUFAs (EPA and DHA) were markedly reduced in MI tissue (Fig. 1B). LSEA demonstrated significant reductions in phosphatidylcholine (PC), phosphatidylethanolamine (PE), phosphatidylinositol (PI), and plasmenyl PE (plasmalogen), whereas TAG levels were significantly increased (Fig. 1C). Other phospholipid classes, including bis(monoacylglycero)phosphate (BMP), phosphatidylglycerol (PG), and phosphatidylserine (PS), also tended to be lower, suggesting globally reduced phospholipid levels. Consistent with the OPLS-DA results, TAG was significantly increased in MI (Fig. 1C).

To mine macro-level structural shifts across the myocardial lipidome, overall acyl-chain length and saturation patterns were evaluated across major structural categories using lipidr, a dedicated software tool for data mining and exploratory visualization of lipidmics datasets [4]. Lipid classes with two fatty acyl chains (phospholipids, sphingolipids, and DAG) were generally reduced in the MI group. Differences between groups were more pronounced in lipids with shorter chain lengths, with C30 species (e.g., C14:0/C16:0) showing the greatest change (Fig. 2A, left). Analysis of total unsaturated bonds showed a biphasic pattern with a peak at three unsaturated bonds, indicating lower levels of both saturated and PUFA-containing lipids in MI (Fig. 2A, right).

**Fig. 1:**
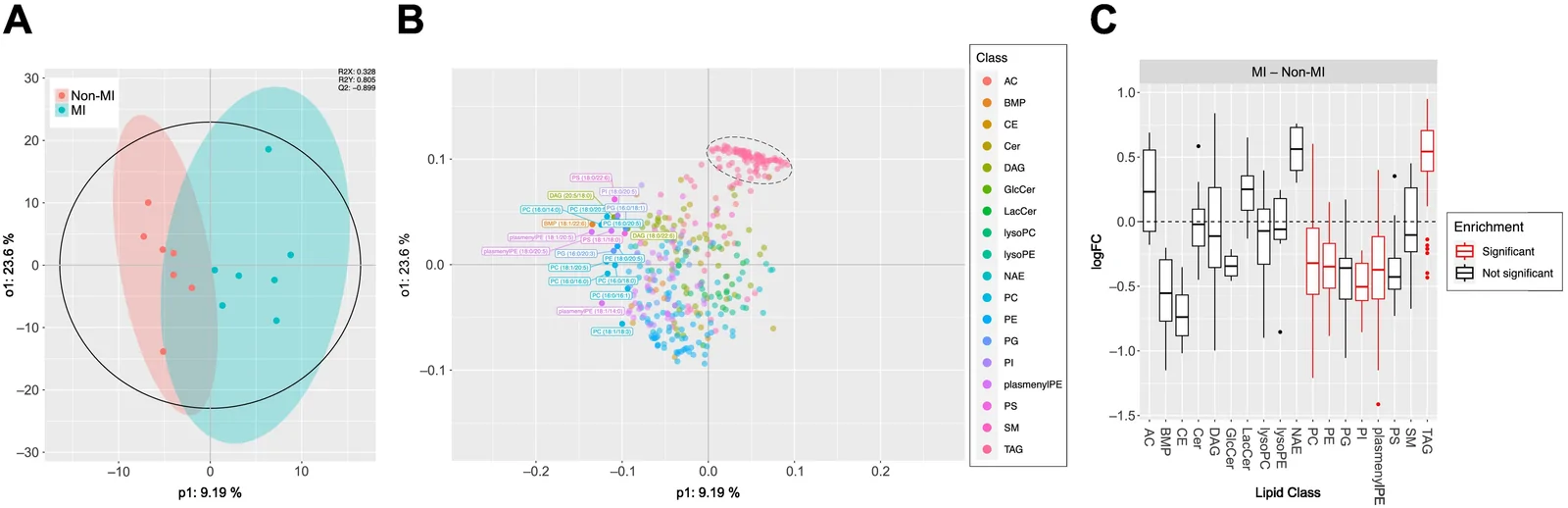
Distinct lipid profiles in LAA tissue from patients with MI. LAA samples were obtained from 14 independent patients (non-MI, *n* = 7; MI, *n* = 7); each n represents one patient-derived biological replicate. (A) OPLS-DA score plot. Circles indicate the 95% confidence limits. (B) OPLS-DA loading plot. The top 20 lipid species are highlighted. The cluster of TAG species is indicated by the dashed circle. (C) LSEA of differentially abundant lipid classes. Lipid species were ranked by log2 fold change (logFC), and class-level enrichment was assessed using fgsea. Benjamini–Hochberg FDR < 0.05 was considered significant, with significant lipid classes highlighted in red. Box plots show the median, interquartile range, and whiskers extending to 1.5 times the interquartile range. AC: acylcarnitine; BMP: bis(monoacylglycero)phosphate; CE: cholesterol ester; Cer: ceramide; DAG: diacylglycerol; GlcCer: glucosylceramide; LacCer: lactosylceramide; logFC: log fold change; lysoPC: lysophosphatidylcholine; lysoPE: lysophosphatidylethanolamine; NAE: N-acylethanolamine; PC: phosphatidylcholine; PE: phosphatidylethanolamine; PG: phosphatidylglycerol; PI: phosphatidylinositol; plasmenylPE: plasmenyl phosphatidylethanolamine (plasmalogen); PS: phosphatidylserine; SM: sphingomyelin; TAG: triacylglycerol. (For interpretation of the references to color in this figure legend, the reader is referred to the web version of this article.)

**Fig. 2:**
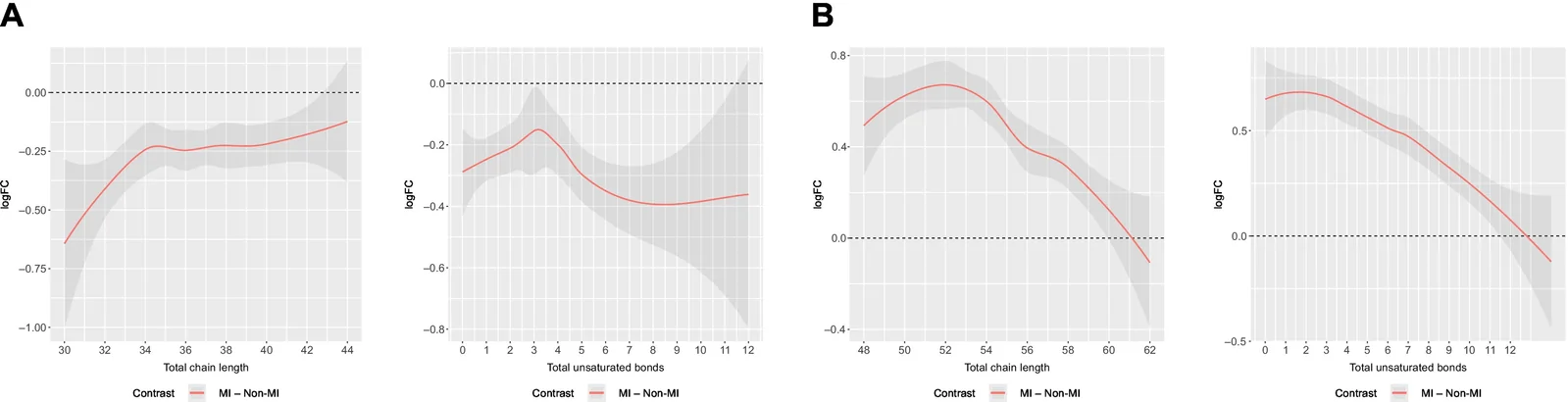
Altered lipid chain length and unsaturation patterns in LAA tissue from patients with MI. LAA samples were obtained from 14 independent patients (non-MI, *n* = 7; MI, *n* = 7); each n represents one patient-derived biological replicate. (A) Lipid chain analysis of lipid classes with two fatty acyl chains. Trend plots show log2 fold change (MI vs. non-MI) according to total chain length (left) and total number of unsaturated bonds (right). (B) Lipid chain analysis of lipid species with three fatty acyl chains (TAG). Trend plots show log2 fold change according to total chain length (left) and total number of unsaturated bonds (right). The shaded areas indicate 95% confidence intervals around the trend lines. No inferential statistical test was applied to these exploratory trend plots.

In contrast, lipid classes with three fatty acyl chains, namely TAG, were overall increased in the MI group (Fig. 2B, left). TAG species with C52 and one or two unsaturated bonds showed the most prominent increases. As chain length and unsaturation increased, TAG levels tended to approach those of the non-MI group (Fig. 2B, right). These findings suggest that TAG species abundant in lipid droplets (e.g., C16:0/C18:1/C18:1) accumulate in the LAA of MI patients, whereas PUFA-rich TAG species (e.g., C20:4/C20:4/C22:6) do not.

This study demonstrates a distinct shift in myocardial lipid composition in patients with MI, characterized by TAG accumulation and depletion of multiple phospholipid classes in LAA tissue. These findings provide tissue-level evidence of myocardial lipid remodeling associated with MI.

The increased TAG accumulation observed in MI patients likely reflects altered myocardial lipid metabolism. Lipid droplets have been identified in infarcted myocardium [5], and increased myocardial TAG levels have been reported in patients with heart failure using ^1^H cardiac magnetic resonance spectroscopy [6]. Excess lipid storage in cardiomyocytes has been linked to impaired cardiac function. TAG metabolism is tightly regulated by adipose triglyceride lipase (ATGL), and disruption of ATGL activity results in marked myocardial lipid accumulation and severe cardiac dysfunction [7]. Although the present study did not evaluate lipid metabolic regulators such as ATGL, these observations suggest that altered TAG uptake, storage, or oxidation may contribute to lipid accumulation in MI myocardium.

In addition to TAG accumulation, we observed marked reductions in phospholipid species containing omega-3 PUFAs and myristic acid, together with decreased levels of major phospholipid classes including PC, PE, PI, and plasmalogens. Epidemiological studies have shown that omega-3 fatty acid intake is associated with reduced cardiovascular mortality and MI risk [8] and that myristic acid intake is inversely associated with MI incidence [9]. Experimental studies further indicate that omega-3 fatty acids modify myocardial membrane phospholipid composition [10]. Because PC and PE are key membrane phospholipids involved in cellular signaling and inflammatory regulation [11], and plasmalogens function as endogenous antioxidants that protect against lipid peroxidation [12], reductions in these lipids may increase myocardial susceptibility to oxidative stress and metabolic dysfunction.

However, the observed lipid alterations may not be attributable solely to prior MI. Underlying chronic cardiac disease may also contribute to myocardial lipid remodeling, as experimental evidence suggests that impaired fatty acid oxidation can be associated with lipid accumulation in the left atrial appendage [13]. Thus, the present findings should be interpreted as MI-associated rather than MI-specific lipid remodeling. The mechanisms underlying these alterations remain unclear but may involve increased fatty acid uptake, enhanced TAG synthesis, impaired lipid degradation, or dysregulated phospholipid remodeling, potentially contributing to myocardial lipotoxicity.

This study has several limitations. First, it was conducted at a single center with a relatively small sample size, and lipidomic analysis was performed in LAA tissue obtained during cardiac surgery, whereas MI-related remodeling primarily occurs in the left ventricle. Second, the non-MI group consisted of patients with stable coronary artery disease rather than healthy individuals; therefore, underlying coronary artery disease and associated metabolic alterations may have influenced lipid profiles in both groups. In addition, differences in statin use and circulating triglyceride levels between the groups may have influenced the observed myocardial TAG profile [14]. Given the small sample size, these potential medication- and metabolic-related confounders could not be adequately adjusted for and should be considered when interpreting the findings. Lipid quantifications were normalized to tissue wet weight rather than dry weight. While wet-weight normalization is a standard approach in human tissue lipidomics to prevent oxidation of labile lipid species (such as polyunsaturated fatty acids and plasmalogens) during lyophilization and to preserve precious clinical samples, potential variations in tissue hydration status between groups could not be entirely ruled out. Despite these limitations, the present findings highlight a characteristic imbalance in myocardial lipid composition in MI and suggest that LC–MS–based lipidomic profiling of human myocardial tissue may provide insights into the metabolic mechanisms underlying MI and lipid-related cardiovascular disease.

## CRediT authorship contribution statement

**Tomoyuki Shiozawa:** Supervision, Investigation, Formal analysis. **Tomoyasu Kadoguchi:** Writing – original draft, Funding acquisition, Formal analysis. **Hyeon-Cheol Lee-Okada:** Resources, Funding acquisition, Formal analysis, Data curation. **Kazunori Shimada:** Supervision, Conceptualization. **Satoshi Matsushita:** Project administration, Methodology. **Takehiko Yokomizo:** Supervision, Funding acquisition. **Tetsuro Miyazaki:** Resources. **Tatsuro Aikawa:** Resources. **Hidemori Hayashi:** Resources. **Satoru Suwa:** Resources. **Atsushi Amano:** Project administration, Methodology. **Yuji Nakazato:** Resources. **Hiroyuki Daida:** Supervision. **Minoru Tabata:** Resources, Methodology. **Naoshi Fukui:** Resources. **Nobuho Tanaka:** Resources. **Hirotaka Tsuno:** Resources. **Tohru Minamino:** Supervision, Resources, Investigation.

## Funding

This work was supported by 10.13039/501100001700JSPS KAKENHI (no. 19K18192 to T.S.; 18K17935 to T.K.; 15H05904, 21H04798, 24H00601 to T.Y.; 15H06600, 18K16246, and 21K08565 to H-C.L-O.).

## Declaration of competing interest

The authors declare that they have no known competing financial interests or personal relationships that could have appeared to influence the work reported in this paper.

## Appendix A Supplementary data

Supplemental Table 1 Baseline characteristics of the patients after propensity score matching. Data are presented as mean ± SD for continuous variables and n (%) for categorical variables. Continuous variables were compared using unpaired *t*-tests or Mann–Whitney *U* tests, as appropriate, after assessment of normality using the Shapiro–Wilk test. Categorical variables were compared using Pearson's chi-square test. *Significant difference from the non-MI group (*p* < 0.05). AF: atrial fibrillation; BMI: body mass index; BNP: B-type natriuretic peptide; BP: blood pressure; BUN: blood urea nitrogen; CKD: chronic kidney disease; CRP: C-reactive protein; Cre: creatinine; eGFR: estimated glomerular filtration rate; HbA1c: glycated hemoglobin A1c; HDL: high-density lipoprotein; LDL: low-density lipoprotein; LVEF: left ventricular ejection fraction; NYHA: New York Heart Association; TC: total cholesterol; TG: triglyceride.

Supplemental Dataset Eighteen lipid classes identified in LAA tissue using wide-target lipidomics analysis.

## References

[1] Lee Hyeon-Cheol, Yokomizo Takehiko (2018). Applications of mass spectrometry-based targeted and non-targeted lipidomics. Biochem Biophys Res Commun.

[2] Kohno S., Keenan A.L., Ntambi J. (2018). Lipidomic insight into cardiovascular diseases. Biochem Biophys Res Commun.

[3] Ri K., Lee-Okada H.C., Yokomizo T. (2022). Omega-6 highly unsaturated fatty acids in Leydig cells facilitate male sex hormone production. Commun Biol.

[4] Mohamed A., Molendijk J., Hill M. (2020). Lipidr: a software tool for data mining and analysis of lipidomics datasets. J Proteome Res.

[5] Hashani M., Witzel H.R., Pawella L.M. (2018). Widespread expression of perilipin 5 in normal human tissues and in diseases is restricted to distinct lipid droplet subpopulations. Cell Tissue Res.

[6] Mahmod M., Pal N., Rayner J. (2018). The interplay between metabolic alterations, diastolic strain rate and exercise capacity in mild heart failure with preserved ejection fraction: a cardiovascular magnetic resonance study. J Cardiovasc Magn Reson.

[7] Da Dalt L., Cabodevilla A.G., Goldberg I.J. (2023). Cardiac lipid metabolism, mitochondrial function, and heart failure. Cardiovasc Res.

[8] Khan S.U., Lone A.N., Khan M.S. (2021). Effect of omega-3 fatty acids on cardiovascular outcomes: a systematic review and meta-analysis. EClinicalMedicine.

[9] Praagman J., Vissers J.E., Mulligan A.A. (2019). Consumption of individual saturated fatty acids and the risk of myocardial infarction in a UK and a Danish cohort. Int J Cardiol.

[10] O’Shea K.M., Khairallah R.J., Sparagna G.C. (2009). Dietary omega-3 fatty acids alter cardiac mitochondrial phospholipid composition and delay Ca2+-induced permeability transition. J Mol Cell Cardiol.

[11] O’Connell T.D., Mason R.P., Budoff M.J. (2020). Mechanistic insights into cardiovascular protection for omega-3 fatty acids and their bioactive lipid metabolites. Eur Heart J Suppl.

[12] Wallner S., Schmitz G. (2011). Plasmalogens the neglected regulatory and scavenging lipid species. Chem Phys Lipids.

[13] Bai F., Liu Y., Tu T. (2019). Metformin regulates lipid metabolism in a canine model of atrial fibrillation through AMPK/PPAR-alpha/VLCAD pathway. Lipids Health Dis.

[14] Aslani S., Razi B., Imani D. (2023). Effect of statins on the blood lipid profile in patients with different cardiovascular diseases: a systematic review with meta-analysis of randomized clinical trials. Curr Med Chem.

