# Supplementary material for: Myocardial lipid remodeling in patients with myocardial infarction undergoing cardiac surgery
Source: J Mol Cell Cardiol Plus. 2026 Oct 7;18:100875. doi: 10.1016/j.jmccpl.2026.100875 (PMC13698078; doi:10.1016/j.jmccpl.2026.100875)
Supplement: Supplementary file 1 [file mmc1.docx]

**Supplemental Table 1. Baseline characteristics of the patients after propensity score matching.**

|  | **Non-MI** | **MI** | **p-value** |
| --- | --- | --- | --- |
| n | 7 | 7 |  |
| Age, years | 67.4 ± 10.6 | 70.0 ± 8.7 | 0.73 |
| Male sex (%) | 6 (86) | 6 (86) | 1.00 |
| BMI, kg/m^2^ | 24.3 ± 2.9 | 24.8 ± 1.8 | 0.73 |
| Hypertension (%) | 6 (86) | 4 (57) | 0.23 |
| Diabetes (%) | 2 (29) | 2 (29) | 1.00 |
| Dyslipidemia (%) | 6 (86) | 5 (71) | 0.51 |
| CKD (%) | 3 (43) | 1 (14) | 0.23 |
| Hemodialysis (%) | 1 (14) | 1 (14) | 1.00 |
| Smoking history (%) | 4 (57) | 4 (57) | 1.00 |
| SBP, mmHg | 123 ± 11 | 136 ± 33 | 0.36 |
| DBP, mmHg | 65 ± 6 | 80 ± 25 | 0.16 |
| HR, bpm | 70.4 ± 12.1 | 78.7 ± 18.8 | 0.35 |
| NYHA ≥ 3 (%) | 1 (14) | 1 (14) | 1.00 |
| LVEF (%) | 52.6 ± 13.8 | 48.1 ± 11.9 | 0.53 |
| Postoperative AF (%) | 1 (14) | 3 (43) | 0.23 |
| Statins (%) | 6 (86) | 2 (29) | 0.03* |
| Beta blockers (%) | 2 (29) | 3 (43) | 0.58 |
| Hemoglobin, g/dL | 12.4 ± 2.0 | 13.7 ± 1.5 | 0.22 |
| Albumin, g/dL | 3.8 ± 0.4 | 4.0 ± 0.3 | 0.58 |
| BUN, mg/dL | 24.3 ± 15.3 | 16.9 ± 6.5 | 0.26 |
| Cre, mg/dL | 3.55 ± 4.86 | 1.61 ± 2.28 | 0.36 |
| eGFR,  mL/min/1.73 m^2^ | 55.3 ± 39.4 | 63.5 ± 30.3 | 0.69 |
| TG, mg/dL | 75 ± 21 | 146 ± 78 | 0.04* |
| TC, mg/dL | 152 ± 32 | 172 ± 45 | 0.36 |
| HDL, mg/dL | 52 ± 17 | 43 ± 7 | 0.20 |
| LDL, mg/dL | 81 ± 28 | 100 ± 46 | 0.36 |
| LDL/HDL | 1.67 ± 0.69 | 2.39 ± 1.21 | 0.20 |
| HbA1c (%) | 6.3 ± 1.1 | 6.5 ± 1.0 | 0.82 |
| CRP, mg/dL | 0.29 ± 0.36 | 0.27 ± 0.34 | 0.94 |
| BNP, pg/mL | 511 ± 855 | 343 ± 410 | 0.65 |

Data are presented as mean ± SD for continuous variables and n (%) for categorical variables. Continuous variables were compared using unpaired t-tests or Mann–Whitney U tests, as appropriate, after assessment of normality using the Shapiro–Wilk test. Categorical variables were compared using Pearson’s chi-square test. *p < 0.05.

AF, atrial fibrillation; BMI, body mass index; BNP, B-type natriuretic peptide; BUN, blood urea nitrogen; CKD, chronic kidney disease; CRP, C-reactive protein; Cre, creatinine; DBP, diastolic blood pressure; eGFR, estimated glomerular filtration rate; HbA1c, glycated hemoglobin A1c; HDL, high-density lipoprotein; HR, heart rate; LDL, low-density lipoprotein; LVEF, left ventricular ejection fraction; MI, myocardial infarction; NYHA, New York Heart Association; SBP, systolic blood pressure; TC, total cholesterol; TG, triglyceride.
